# Long-amplicon MinION-based sequencing study in a salt-contaminated twelfth century granite-built chapel

**DOI:** 10.1007/s00253-022-11961-8

**Published:** 2022-05-21

**Authors:** Jelena Pavlović, Pilar Bosch-Roig, Magdalena Rusková, Matej Planý, Domenico Pangallo, Patricia Sanmartín

**Affiliations:** 1 Institute of Molecular Biology, Slovak Academy of Sciences, Dúbravská cesta 21, 845 51 Bratislava, Slovakia; 2 Instituto Universitario de Restauración del Patrimonio, Universitat Politècnica de València, 46022 Valencia, Spain; 3 Caravella, s.r.o., Tupolevova 2, 851 01 Bratislava, Slovakia; 4 Departamento de Edafoloxía e Química Agrícola, Facultade de Farmacia, Universidade de Santiago de Compostela, 15782 Santiago de Compostela, Spain; 5 CRETUS, Universidade de Santiago de Compostela, Santiago de Compostela, Spain

**Keywords:** Long amplicons, MinION sequencing, Salt contamination, Stone, *Batrachochytrium*, Bio-desalination

## Abstract

**Abstract:**

The irregular damp dark staining on the stonework of a salt-contaminated twelfth century granite-built chapel is thought to be related to a non-homogeneous distribution of salts and microbial communities. To enhance understanding of the role of microorganisms in the presence of salt and damp stains, we determined the salt content and identified the microbial ecosystem in several paving slabs and inner wall slabs (untreated and previously bio-desalinated) and in the exterior surrounding soil. Soluble salt analysis and culture-dependent approaches combined with archaeal and bacterial 16S rRNA and fungal ITS fragment as well as with the functional genes *nirK*, *dsr*, and *soxB* long-amplicon MinION-based sequencing were performed. State-of-the-art technology was used for microbial identification, providing information about the microbial diversity and phylogenetic groups present and enabling us to gain some insight into the biological cycles occurring in the community key genes involved in the different geomicrobiological cycles. A well-defined relationship between microbial data and soluble salts was identified, suggesting that poorly soluble salts (CaSO_4_) could fill the pores in the stone and lead to condensation and dissolution of highly soluble salts (Ca(NO_3_)_2_ and Mg(NO_3_)_2_) in the thin layer of water formed on the stonework. By contrast, no direct relationship between the damp staining and the salt content or related microbiota was established. Further analysis regarding organic matter and recalcitrant elements in the stonework should be carried out.

**Key points:**

• *Poorly (CaSO*_*4*_*) and highly (Ca(NO*_*3*_*)*_*2*_*, Mg(NO*_*3*_*)*_*2*_*) soluble salts were detected*

• *Halophilic and mineral weathering microorganisms reveal ecological impacts of salts*

• *Microbial communities involved in nitrate and sulfate cycles were detected*

**Supplementary Information:**

The online version contains supplementary material available at 10.1007/s00253-022-11961-8.

## Introduction

Stone monuments provide habitats for multispecies microbial communities, including bacteria, fungi, lichens, algae, and archaea (Hoppert et al. 2004; Gadd 2017). The presence of microbes on heritage stonework is generally considered negative owing to the physical and/or chemical damage that they cause via biodeterioration mechanisms. However, the presence of these communities can also have negligible effects, such as surface deposition with no substrate interaction and only an esthetic impact (Sanmartín et al. 2020), and even positive effects, such as bioprotection, biomineralization, and bio-desalination phenomena (Kembel et al. 2014; Pinna 2014; Gadd 2017; Schröer et al. 2021; Ortega-Morales and Gaylarde 2021; Bosch-Roig et al. 2021). As geomicrobial agents on the built environment, stone colonizers are involved in elemental cycling, rock transformation, soil formation, organic matter decomposition, and cycling of elements, among other processes (Barton and Northup 2007; Gadd 2017) .

The biological population associated with stone-built structures is determined by a combination of factors such as environmental conditions (e.g., humidity, temperature, light, and pollution), architectural design, the chemical and mineralogical composition, and petrophysical properties of the stone (e.g., roughness and pore structure) and anthropogenic influences (e.g., human occupancy and restoration processes) (Gorbushina and Broughton 2009; Gadd 2017). The presence of salts also affects stone-associated microbial communities, which are often rich in highly specialized microorganisms such as halophilic microorganisms (Schabereiter-Gurtner et al. 2004). Indeed, desalination treatments may affect the endogenous microbial communities (Caneva et al. 2005).

Salt contamination is widespread and very difficult to eradicate from stone monuments. Salts can cause both esthetic and physical damage, and salt contamination is one of the most important factors involved in the deterioration of stone monuments, leading to important cultural and economic losses (Freedland 1999; Germinario and Oguchi 2021). The behavior of salt on stone-built cultural heritage surfaces is a complex phenomenon and involves multiple variables, such as salt type, salt combinations and quantity (supersaturation), substrate properties (porous size), and environmental factors (temperature and humidity). In addition, there are multiple sources of soluble salts in stone material (Freedland 1999; Barton and Northup 2007), such as the building material itself (e.g., manufacturing process), water (e.g., seawater), atmospheric pollution (e.g., acid rain), modern interventions/treatments (e.g., restoration products), and microorganisms (e.g. ammonia-oxidizing bacteria). Furthermore, salt contamination, often related to the presence of water, is an important factor influencing microbial growth (Hoppertet al. 2004; Dedesko and Siegel 2015).

The present study concerns the salt-contaminated twelfth century granite-built Cristo Chapel of the Santa María de Conxo Monastery in Santiago de Compostela (NW Spain). The stonework is affected by irregular, damp-to-touch dark staining that may be caused by a non-homogeneous distribution of salts and biological factors. To enhance understanding of the role of microorganisms in the presence of salts and in the staining, stonework (including chapel and church paving slabs and the slabs in the inner wall of the chapel) and the soil surrounding the chapel were analyzed to determine the salt content and identify the microbes present. Different strategies were used to identify culture-dependent and culture-independent microorganisms (and thus identify non-culturable microorganisms) (Schröer et al. 2020; Elert et al. 2021). High-throughput sequencing, which has revolutionized the analysis of microbiota on cultural heritage objects (Marvasi et al. 2019), was also used. Among the sequencing approaches, the third-generation MinION sequencing platform (Oxford Nanopore Technologies, ONT, Oxford, UK), also used in this work, is an affordable device that can be used to identify the microorganisms present in samples, therefore enabling detailed study of microbial communities (Bosch-Roig and Sanmartín 2021; Pavlović et al. 2021). One of the advantages of the MinION sequencing approach is the ability of the technique to generate and analyze long reads (including long amplicons), which can enhance identification of the microbiota of interest (Pavlović et al. 2021).

A high presence of salt related to the microbial communities on the pavement causing the damp dark staining is the hypothetical premise of the present study. To address it, analysis of soluble salts was coupled with a culture-dependent analysis and a long-amplicon MinION-based sequencing strategy focusing on archaeal and bacterial 16S rRNA, fungal ITS fragment, and the *nirK* (nitrite reductase), *dsr* (dissimilatory sulfite reductase), and *soxB* (sulfite oxidase) functional genes.

## Materials and methods

### Site description

The Santa María de Conxo Monastery, located in Santiago de Compostela (UNESCO World Heritage City since 1985, capital of Galicia, NW Spain) is a baroque complex of seventeenth century buildings, constructed around a Romanesque twelfth century chapel called the Cristo Chapel. The monastery complex includes a direct connection between the chapel, the church, and the cemetery. The walls and the paving of the Cristo Chapel and the church consist of granite slabs. The paving slabs of the Cristo Chapel (occupying an area of 233 m^2^) are strongly affected by salt contamination and irregular damp dark staining. In order to eliminate or at least reduce the dark staining, different treatments were applied between 2013 and 2020, including architectural interventions and bio-desalination treatments by the addition of live *Pseudomonas stutzeri* denitrifying bacteria (García Morales et al. 2016; Bosch-Roig et al. 2019, 2021). Although the treatments/interventions reduced the damp dark staining, it is still present.

### Sampling

**Fig. 1:**
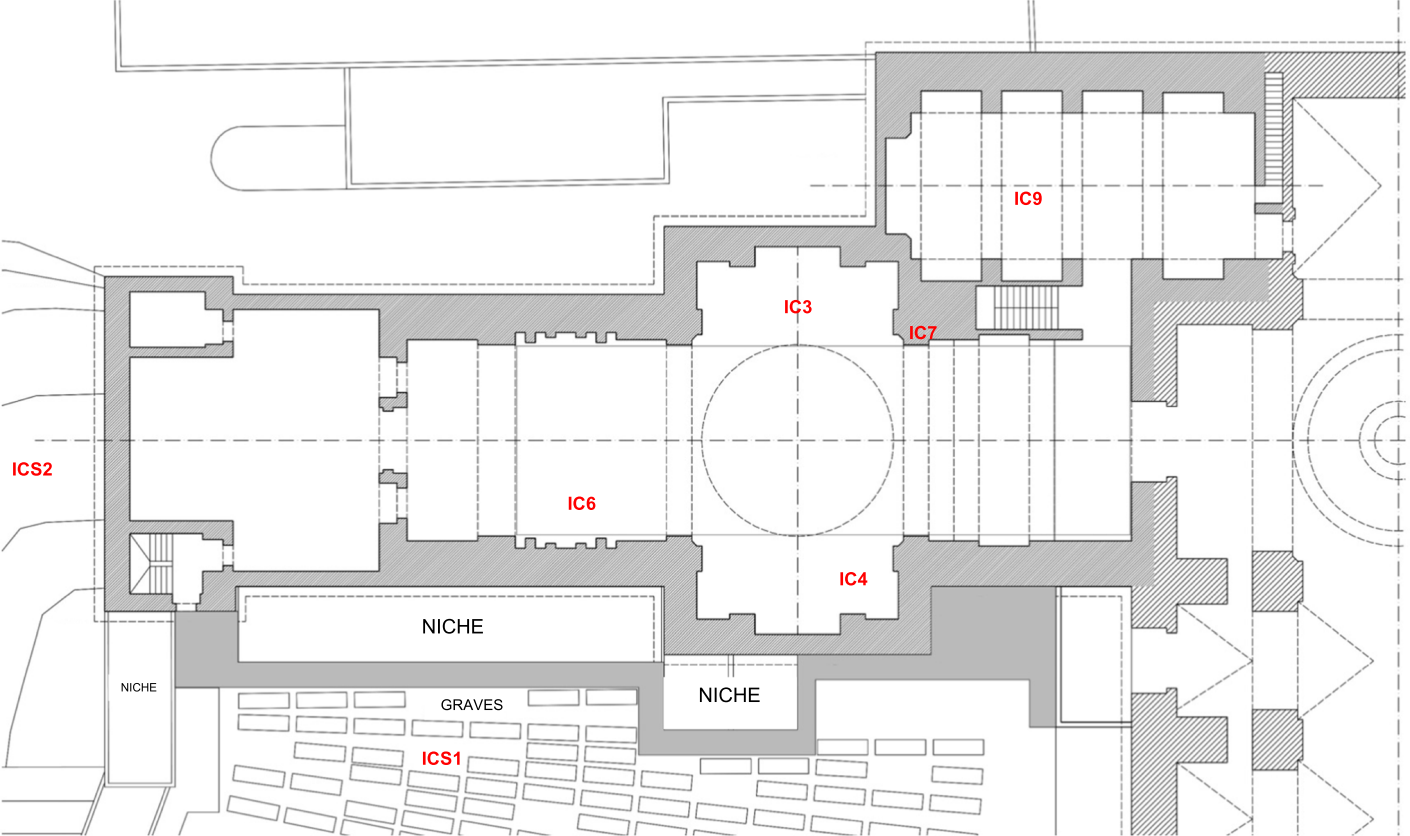
Plan of the Santa María de Conxo Monastery (provided by Alicia Noia and Lourdes Pérez from the Consorcio de Santiago) showing the seven sampling points in red: IC3, IC4, IC6, IC7, IC9, ICS1, and ICS2

The sampling methodology was designed to evaluate both the salt content and the microbiome in order to study their relationship and their potential involvement in causing the damp staining. Sampling was carried out on January 21, 2020, i.e., one to three months after a bio-desalination treatment. Seven sampling points were selected, including damp to touch dark patches and dry darkened areas, bio-desalinated and untreated areas, and four different locations (chapel paving, church paving, chapel inner wall, and the soil surrounding the chapel). Of these, five samples (IC3, IC4, IC6, IC7, IC9) were taken from inside and two samples (ICS1, ICS2) from the soil outside the building (Fig. 1, Table 1). Samples IC3 and IC4 were taken respectively from two damp dark patches on chapel paving slabs, with different degrees of discoloration relative to the rest of the chapel paving. Thus, IC3 was from a darker, more heterogenous slab with a southern orientation, and IC4 was from a lighter, more homogeneous slab, with a northern orientation. Both areas had been treated by bio-desalination with the bacterium *P. stutzeri*. This treatment involves the addition of live *P. stutzeri* directly to the stone surface, followed by the application of ground 2% agar gel (as a delivery system and source of humidity) and of an electric heating mat (26 ± 4 °C for 48 h) (as described in Bosch-Roig et al. 2019, 2021). After the treatment, the thermal and delivery systems are removed, and the surface is cleaned with distilled water and a sponge. Likewise, the exogenous introduction of *P. stutzeri* and its permanence is checked with CFU (colony-forming unit) content, not obtaining significant differences with respect to the beginning (before bio-desalination) (Bosch-Roig et al. 2021). Sample IC6 was taken from a damp dark patch of chapel paving slab that had been partially treated (unfinished treatment) by bio-desalination, in which the *P. stutzeri* had been added, but the agar gel and heat had not been applied and the final cleaning step with water had not been carried out. This sample was taken to evaluate the potential change in microbial diversity on the stone slab during the bio-desalination treatment. Sample IC7 was taken from a slab from the inner wall of the chapel, not affected by damp staining and not treated by bio-desalination. Sample IC9 was taken from an untreated church paving slab affected by damp dark staining, for purposes of comparison. Finally, two samples of soil surrounding the chapel were collected: from an area adjoining the cemetery (ICS1) and from an area not adjoining the cemetery (ICS2).

**Table 1:**
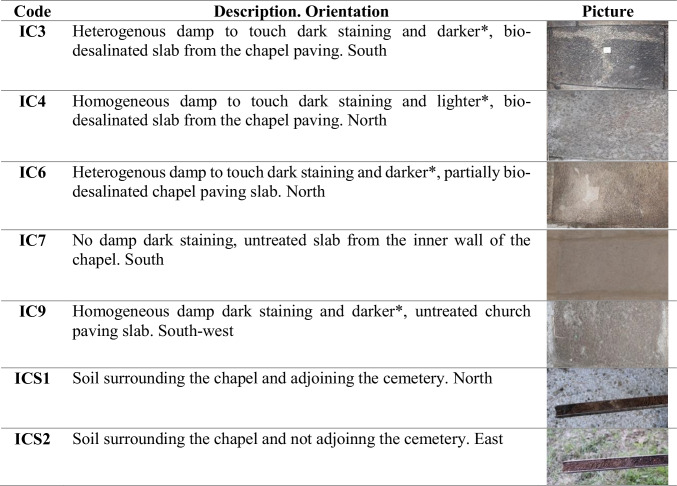
Visual description and details of the seven sampling points

^*^Relative to the chapel paving slabs

For each sampling point inside the building, a granite slab was selected and the entire surface was scratched with a sterile scalpel to yield approximately 0.3–0.8 g of surface deposit, which was placed in sterile 50-mL plastic tubes. For each sampling point outside the building, composite soil samples were prepared by combining four subsamples from the same area. The soil subsamples were collected within an area of ~ 1 m diameter, to a depth of 50 cm, with an ethanol-sterilized soil probe or hand auger. The subsamples were mixed together in a sterile 500-mL glass container, which was vigorously shaken to produce a homogenous sample. In both cases, the samples were transported to the laboratory, where each of the seven samples was divided into four subsamples for soluble salt analysis, DNA-based analysis in the MiniON device, and culture-dependent techniques. One subsample was reserved for possible further analyses.

### Material characterization: soluble salt content and soil texture

The soluble salts were extracted from all samples by a simple shaking-assisted method with water, for 24 h (100 mg of sample in 100 mL of MilliQ water), following an optimized methodology based on UNE-EN 16, 455:^2016^. The ionic composition was determined in an ion chromatographer (Metrohm 930 Compact IC Flex, Riverview, FL, USA). The results are expressed as milligram of ion (anion or cation) per gram of sample. The texture of soils was determined according to a standard procedure, in which the soil samples were air-dried and sieved through a 2-mm-mesh-size sieve, and any plant roots were removed. Particle size distribution was determined by wet sieving and the pipette method (Gee and Bauder 1986) after the removal of organic matter and iron oxides.

### Isolation of microorganisms

About 20 mg of each sample from the monastery (IC3, IC4, IC6, IC7, IC9) and 50 mg of each sample from the surrounding soil (ICS1, ICS2) were placed in plastic tubes with 2 mL of physiological solution and stirred briefly. The seven suspensions were serially diluted to 1 × 10^−8^, and 100 µL of each dilution was then plated in 9 different agar media including general isolation media and specific isolation media (according to the salt concentration present on the pavement) without addition of any colorant, to isolate the microbes present on the slabs. For the isolation of fungi, 3 different specific agar media were used: malt extract agar (MEA; Himedia, Mumbai, India); MEA with 3% NaCl and 2% Mg_2_SO_4_; and dichloran-glycerol (DG-18; Merck, Darmstadt, Germany). For the isolation of bacteria, 6 different specific agar media were used: Reasoner’s 2A (R2A; Himedia); R2A with 3% NaCl and 2% Mg_2_SO_4_; ammonia oxidation agar (AOA medium containing (NH_4_)_2_SO_4_, 0.5 g; KH_2_PO_4_, 0.2 g; MgSO_4_.7H_2_0, 0.2 g; CaCI_2_.2H_2_0, 0.02 g; distilled water, 1 l, with pH of adjusted to 8.2 with 0.1 N NaOH, Sarathchandra 1979); denitrification screening agar (DSA, including KNO_3_ 1 g; CaCl_2_ 0.2 g; KH_2_PO_4_ 1 g; FeCl_3_·6H_2_O 0.5 g; MgSO_4_·7H_2_O 1 g; sodium succinate 8.6 g; agarose 21 g; distilled water 1 l, the final pH of the medium was adjusted to 7.0 ± 0.2, Shao et al. 2016), *Thiobacillus* agar (TBA, for the isolation of sulfate-oxidizing bacteria, containing (NH_4_)_2_SO_4_ 0.4 g; MgSO_4_ 7H_2_O 0.5 g; CaCl_2_ 0.25 g; KH_2_PO_4_ 4 g; FeSO_4_ 0.01 g; Na_2_S_2_O_3_ 5 g; agar 12.5 g; distilled water 1 l, the final pH of the medium was adjusted to 4.0–4.5; Starosvetsky et al. 2013); and actinomycete isolation agar (AIA; Himedia). All plates were incubated at room temperature (24–26 °C) for 3 weeks. After the selection of pure colonies, based on their macro-morphology and color, the fungi were maintained on MEA and the bacteria were maintained on R2A plates.

### Specific plate assays

The isolated bacteria and fungi were tested for different activities/capacities using specific agar plate assays.

For screening microorganisms involved in nitrate and sulfate cycles, the following media were used: AOA with phenol red (phenol red 7.5 mg/L; Merck, Darmstadt, Germany) for detecting ammonium-oxidizing bacteria; DSA with bromothymol blue (BTB, 1 mL/L; Merck, Darmstadt, Germany) for screening aerobic denitrifiers; TBA with bromocresol green (BCG, 2 mL/L; Merck, Darmstadt, Germany) for detecting sulfate-oxidizing bacteria. All plates were incubated at room temperature and the color change in agar media was monitored over a period of 1–3 weeks. The screening assay is based on the color change of the pH indicator. Screening for the ability to solubilize and precipitate CaCO_3_ was conducted using CaCO_3_ glucose agar (glucose, 10 g; CaCO_3_, 5 g; agar, 15 g, distilled water, 1L, Albertano and Urzì, 1999) and B4 medium (calcium acetate, 2.5 g; yeast extract, 4 g; glucose, 10 g; agar, 15 g; distilled water, 1L, the final pH of the medium was adjusted to 8.0, Boquet et al. 1973) respectively. On CaCO_3_ glucose agar, a clear zone was observed around positive strains, whereas on B4, the positive strains produced crystals. All assays were performed in triplicate 60-mm Petri plates.

### Identification of the microorganisms isolated

Pure bacterial strains were collected from plates, and the DNA was extracted with the DNasy Blood & Tissue Kit (Qiagen, Hilden, Germany) according to the manufacturer’s protocol. The fungal strains were inoculated in Malt Extract Broth (MEB. HiMedia, Maharashtra, India) at 26 °C until growth. The fungal pellets were then separated from the broth by filtration through sterile filter paper. The DNA of the fungal pellets was extracted with the DNeasy Plant Mini Kit (Qiagen, Hilden, Germany) according to the manufacturer’s protocol. For identification of the isolated bacteria by sequencing, the 16S rRNA gene was amplified using the primers 27F (5′–AGA GTT TGA TCC TGG CTC AG-3′) and 685R (5′-TCT ACG CAT TTC ACC GCT AC-3′) according to Lane (1991). The fungal isolates were identified by PCR amplification and by Sanger sequencing of the ITS fragment with the primers ITS1 (5′-TCC GTA GGT GAA CCT GCG G-3′) and ITS4 (5′-TCC TCC GCT TAT TGA TAT GC-3′) according to White et al. (1990). Twenty-five microliters of PCR mixture contained 50 pmol of each primer, 200 µmol/L of dNTP (Life Technologies, Gaithersburg, MD, USA), 1.5 U HotStar Taq plus DNA polymerase (Qiagen), 1 × PCR buffer and 3 µL of the extracted bacterial or fungal DNA. The PCR program consisted of initial denaturation at 94 °C for 5 min, followed by 30 cycles (denaturation at 94 °C for 30 s, annealing at 54 °C for 45 s, extension at 72 °C for 1 min) and a final polymerization step at 72 °C for 8 min. PCR products from bacterial and fungal isolates were purified using ExoSAP-IT (Affymetrix, Cleveland, OH, USA) and sequenced at a commercial facility (Eurofins Genomics, Ebersberg, Germany). The sequences obtained were directly compared with sequences in GenBank by using the BLAST program (http://blast.ncbi.nlm.nih.gov/Blast.cgi) and were subsequently deposited in GenBank under the accession numbers OL423372–OL423396 (bacterial isolates) and OL439055-OL439059 (fungal isolates).

### MinION sequencing

#### DNA extraction and PCR amplification

For MinION sequencing, the total DNA of the seven samples, i.e., five samples from the monastery (IC3, IC4, IC6, IC7, IC9) and two samples from the surrounding soil (ICS1, ICS2) was extracted from the rest of suspensions (whose quantities of material have been described in the “Isolation of microorganisms” section) using the DNeasy PowerSoil Pro extraction kit (Qiagen) following the protocol provided by the producer. Prior to sequencing, total DNA was amplified by specific PCR reactions targeting generic genes for fungal ITS (the same primers and PCR program as in the previous section), for bacterial 16S rRNA (27F: 5′–AGA GTT TGA TCC TGG CTC AG-3′/1492R: 5′–AGA GTT TGA TCC TGG CTC AG-3′) and for archaeal 16S rRNA (Arc344F-mod: 5′-ACG GGG YGC ASS AGK CGV GA-3′/Arch958R-mod: 5′-YCC GGC GTT GAV TCC AAT T-3′) according to Kraková et al. (2016). The presence of microorganisms involved in nitrate and sulfate cycles was established by identifying the functional genes encoding the enzyme nitrite reductase (*nirK*, denitrifying bacteria; nirKC1F: 5′-ATG GCG CCA TCA TGG TNY TNC C-3′/nirKC1R: 5′-TCG AAG GCC TCG ATN ARR TTR TG-3′, according to Wei et al. 2015), the enzyme dissimilatory sulfite reductase (*dsr*, sulfate-reducing bacteria; DSR1Fdeg: 5′-ACS CAY TGG AAR CAC G-3′/DSR4Rdeg: 5′-GTG TAR CAG TTD CCR CA-3′, according to Wagner et al. 1998), the Sox enzyme system for sulfur oxidation (*soxB*, sulfur-oxidizing bacteria; soxB432F: 5′-GAY GGN GGN GAY ACN TGG-3′/soxB1446B: 5′-CAT GTC NCC NCC RTG YTG-3′, according to Petri et al. 2001), and the enzyme ammonia monooxygenase (*amoA*, ammonia-oxidizing bacteria; amoA-1F: 5′-GGG GTT TCT ACT GGT GGT-3′/amoA-2R: 5′-CCC CTC KGS AAA GCC TTC TTC-3′, according to Rotthauwe et al. 1997). The PCR programs used were the same as those applied in the above cited studies (inside the brackets following the primer sequences).

#### Library preparation and sequencing

The sequencing library was prepared according to instructions provided in the protocol for Rapid PCR Barcoding Kit (SQK-RPB004. Oxford Nanopore Technologies, ONT, Oxford, UK), downloaded from the ONT website. The concentration of purified amplicons was determined with a DeNovix QFX Fluorometer (DeNovix Inc., Wilmington, DE, USA) and the DeNovix dsDNA Broad Range Kit (DeNovix). All amplicons were subsequently diluted to appropriate input concentrations ranging between 0.5 and 1.5 ng/µL. Three µL of 1–5 ng template DNA of each sample were used, following slight modifications of the protocol instructions for all the amplicons, except the bacterial 16S rRNA and *dsr* gene in the purification steps performed with AMPure XP beads (Beckman Coulter, Pasadena, CA, USA). For the ITS, archaeal 16S rRNA, *nirK*, and *soxB* amplicons, the previously optimized volume of AMPure beads (60 μL) was used, rather than the recommended 30 µL, to prevent the amplicons from being washed out because of their different, relatively short lengths. Twelve barcoded libraries were pooled in desired ratios to a total molar concentration ranging between 50 and 100 fmoles in 10 µL and ligated with RAP adapters included in the SQK-RPB004 kit (ONT). The prepared libraries were used for loading into the MinION flow cell FLO-MIN 106D R9 Version (ONT). The sequencing was performed in two separate runs (12 libraries for each run). The library features (correlation of samples and amplicons sequenced in each run) are provided in Supplementary material Tables S1 and S2. Sequencing data were split by barcodes with EPI2ME Desktop Agent (ONT). Taxonomic classification and quantitative analysis of the reads derived from bacterial 16S rRNA amplicons were performed using the EPI2ME 16S workflow in EPI2ME Desktop Agent. For the fungal ITS, archaeal 16S rRNA, and other specific markers, the “What’s in my pot” tool (EPI2ME WIMP workflow; ONT) was used. The minimal quality score in both cases was set by default to 7. The percentage of appropriate taxa on the genus taxonomic level was calculated from the total number of classified reads and the number of reads identified as given taxa. The percentage of given taxa was graphically visualized using bar plots. The sequences obtained by the metagenomic analysis are registered and publicly available as BioProject PRJNA767009.

## Results

### Salt content

The soluble ion contents of each of the seven samples are shown in Table 2. The salt content was higher in the samples from inside the building than in the samples from outside of the building, where the total ion content did not exceed 0.198 mg/g soil. This finding can be explained by the coarse texture of the soils (Supplementary material Table S3). The particle size distribution was very similar in both soils, with a major sandy fraction of 72.4% and 69.4%, followed by silt content of 20.7% and 23.0%, and little clay, only 6.9% and 7.5%. In these coarse-textured soils, the loss of mobile particles (such as cations and anions) by leaching is frequent and is greatly favored by the climate of Santiago de Compostela, characterized by high rainfall throughout the year (Martínez-Cortizas and Pérez-Alberti 1999).

**Table 2:** Concentration of soluble ions (mg) per gram rock or soil material from the seven sampling points

Sample	Na^+^	NH_4_^+^	K^+^	Ca^2+^	Mg^2+^	Cl^−^	NO_3_^−^	SO_4_^2−^	PO_4_^3−^	Total
IC3	1.702	0.002	0.177	2.610	0.393	1.250	9.464	1.139	0.083	16.820
IC4	1.952	0.005	0.422	34.177	0.369	1.778	13.869	54.635	0.041	107.248
IC6	0.011	0.450	17.834	0.129	20.671	0.538	23.788	0.973	20.671	85.062
IC7	1.628	0.013	0.961	17.044	0.108	2.026	16.883	19.271	0.024	57.958
IC9	0.563	0.001	0.656	3.060	0.060	0.616	5.743	3.260	0.013	13.972
ICS1	0.017	0.004	0.010	0.029	0.002	0.020	0.012	0.010	0.006	0.110
ICS2	0.013	0.003	0.018	0.122	0.002	0.015	0.008	0.015	0.002	0.198

The building samples with the highest total salt contents were IC4 (107.248 mg/g rock) and IC6 (85.062 mg/g rock), followed by IC7 (57.958 mg/g rock), and the samples with the lowest total salt contents were IC3 (16.820 mg/g rock) and IC9 (13.972 mg/g rock). The most abundant anions were nitrate (NO_3_^−^), with up to 23.788 mg/g of rock in sample IC6, and sulfate (SO_4_^2−^), with up to 54.635 mg/g rock in sample IC4. Phosphate (PO_4_^3−^) was not common, except in sample IC6, with 20.671 mg/g rock. The most abundant cations were calcium (Ca^2+^), with up to 34.177 mg/g rock in sample IC4, magnesium (Mg^2+^), with up to 20.671 mg/g rock in sample IC6, and potassium (K^+^), with up to 17.834 mg/g rock in sample IC6. Thus, the main salts present are expected to be calcium nitrate, potassium nitrate (niter), sodium nitrate (nitratine), calcium sulfate (gypsum), magnesium nitrate, and to a lesser extent sodium chloride (halite) and potassium chloride. Sample IC6 also contained phosphates, probably magnesium phosphate in the form of struvite ((NH_4_)MgPO_4_·6H_2_O), a typical by-product of bacterial activity (Rivadeneyra et al. 1992), and potassium and ammonium phosphates.

### Culture-dependent analysis and properties of isolates

**Fig. 2:**
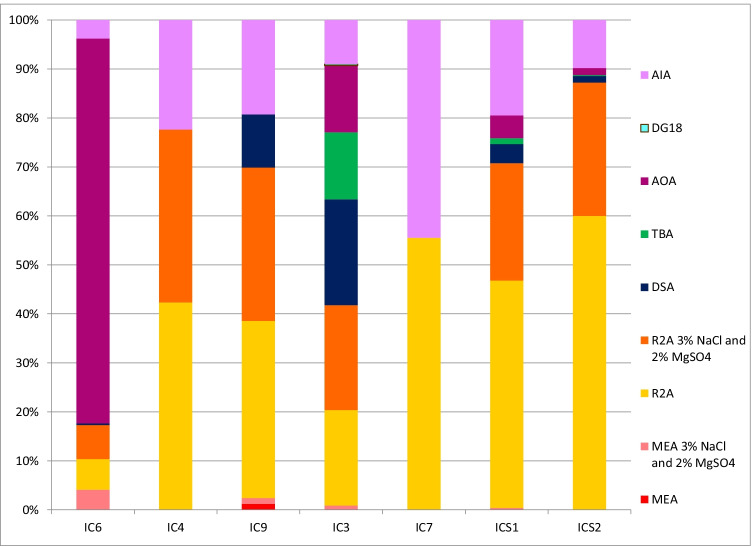
Microorganisms isolated from all samples (monastery and surrounding soil) using the nine different agar media, described in the “Isolation of microorganisms” section. AIA: Actinomycete Isolation Agar, DG18: Dichloran-Glycerol (fungi), AOA: Ammonia Oxidation Agar, TBA: *Thiobacillus* agar, DSA: Denitrification Screening Agar, R2A: Reasoner’s 2A agar, MEA: Malt Extract Agar (fungi)

A concentration in the range 10^1^–10^3^ CFU/mL was evidenced on the agar media used in the culture-dependent analysis. The highest values of CFU/mL (10^3^) were recorded in sample IC3, and the constituent microorganisms also grew in all nine agar media, while the lowest values of CFU/mL (10^1^) were recorded in sample IC7. Clear differences between the microbial communities associated with the stonework of the building and the surrounding soil -and also between the samples of these materials- were observed (Fig. 2). The R2A agar and R2A agar supplemented with NaCl (3%) and MgSO_4_ (2%) were the most effective media for isolating bacteria from all samples, except sample IC6. The few fungi isolated were mainly cultivated on MEA with NaCl (3%) and MgSO_4_ (2%).

A total of 43 morphologically different colonies of microorganisms were isolated from the five sampling points in the monastery: i.e., 13 from IC3, 10 from IC9, 7 from IC4 and from IC7, and 6 from IC6 (Table 3). Identification of the isolates revealed the presence of members of the genus *Pseudomonas* in all samples. The genus *Flavobacterium* was also isolated from samples IC3, IC7, and IC9. Members of the class *Actinomycetes* appeared in each sample and were particularly diverse in sample IC3. Representative members of the classes *Bacilli* and *Alphaproteobacteria* were also isolated. Fungi belonging to the genera *Purpureocillium* (IC6 and IC9), *Penicillium* (IC9), and *Aspergillus* (IC3) were only isolated from samples IC3, IC6, and IC9.

**Table 3:** Identification and capacities/activities of the microorganisms isolated from the five sampling points in the monastery

Sample	Isolates	Morphology	Identification	CaCO_3_ solubilization	CaCO_3_ precipitation	Ammonium oxidation	Denitrification
IC6	B_IC6_A	Milky, circular	MH127765 *Pseudomonas vancouverensis* 99.84%	** + **	**-**	** + + **	** + + **
	B_IC6_D	Pink, raised, oval	LK020736 *Methylobacterium populi* 100%	**-**	**-**	**-**	** + **
	B_IC6_H1	Dark yellow, irregular margins	MF948937 *Pseudomonas* sp. 100%	**-**	**-**	** + + **	** + + **
	B_IC6_L	Pale yellow, circular	LT718481 *Pseudomonas* sp. 99.51%	** + **	**-**	** + + **	** + + **
	B_IC6_N	White with yellow margin	LR596321 *Streptomyces* sp. 100%	**-**	**-**	**-**	** + **
	F_IC6_A1	White, hairy, raised	MT606206 *Purpureocillium* sp. 100%	**-**	**-**	**-**	** + **
IC4	B_IC4_E	Orange, circular colony	MK241856 *Bacillus* sp. 99.84%	** + **	** + **	**-**	**-**
	B_IC4_J	White, filamentous	MT586023 *Bacillus mycoides* 100%	**-**	**-**	** + **	** + **
	B_IC4_N	Bright yellow, circular colony	MG705620 *Corynebacterium* sp. 100%	**-**	** + + **	** + **	** + **
	B_IC4_O	Large light orange colonies, slime	CP054880 *Pseudomonas* sp. 100%	**-**	**-**	** + + **	** + + **
	B_IC4_P	Milky, slime	MH337920 *Rhizobium* sp. 100%	**-**	**-**	** + + **	** + + **
	B_IC4_T	Pinkish, circular	KY992915 *Paenibacillus* sp. 100%	**-**	**-**	**-**	** + **
	B_IC4_W	Pale cream color, regular margins	AY167846 *Janibacter limosus* 100%	** + **	**-**	**-**	** + **
IC9	B_IC9_B	Pink, circular	MN098864 *Sporosarcina aquimarina* 100%	**-**	** + **	**-**	**-**
	B_IC9_C	Yellow, circular, raised	MK670519 *Flavobacterium* sp. 99.17%	**-**	**-**	**-**	**-**
	B_IC9_G	White, small colonies	MH699287 *Streptomyces* sp. 100%	**-**	**-**	** + + **	** + **
	B_IC9_H	Milk, opaque, circular	MK371085 *Pseudomonas* sp. 99.84%	**-**	**-**	** + + **	** + + **
	B_IC9_I	Yellow, slime	MN098866 *Paenarthrobacter nicotinovorans* 100%	**-**	**-**	** + + **	** + + **
	B_IC9_N	White with yellow margin	LR596321 *Streptomyces* sp. 100%	**-**	**-**	**-**	** + **
	B_IC9_S	White, slime	MH549189 *Flavobacterium resistens* 100%	**-**	**-**	**-**	** + + **
	B_IC9_U	Yellow-white, lobate margin	MT102121 *Pseudomonas putida* 100%	** + **	**-**	** + **	** + **
	F_IC9_M	White, hairy, raised	MT529633 *Purpureocillium lilacinum* 100%	**-**	**-**	**-**	**-**
	F_IC9_R	White, irregular	MK450691 *Penicillium decumbens* 100%	**-**	**-**	**-**	**-**
IC3	B_IC3_C	Yellow, circular, raised	MK670519 *Flavobacterium* sp. 99.17%	**-**	**-**	**-**	**-**
	B_IC3_D	Pink, raised, oval	LK020736 *Methylobacterium populi* 100%	**-**	**-**	**-**	** + **
	B_IC3_E	Orange, circular colony	MK241856 *Bacillus* sp. 99.84%	** + **	** + **	**-**	**-**
	B_IC3_H	Milk, opaque, circular	MK371085 *Pseudomonas* sp. 99.84%	**-**	**-**	** + + **	** + + **
	B_IC3_H1	Dark yellow, irregular margins	MF948937 *Pseudomonas* sp. 100%	**-**	**-**	** + + **	** + + **
	B_IC3_I	Yellow, slime	MN098866 *Paenarthrobacter nicotinovorans* 100%	**-**	**-**	** + + **	** + + **
	B_IC3_L	White, irregular margins	KJ816785 *Brevibacterium linens* 100%	** + **	** + + **	**-**	** + **
	B_IC3_O	Big light orange colonies, slime	CP054880 *Pseudomonas* sp. 100%	**-**	**-**	** + + **	** + + **
	B_IC3_S	White, slime	MH549189 *Flavobacterium resistens* 100%	**-**	**-**	**-**	** + + **
	B_IC3_S1	Pale yellow, irregular margins	JQ291594 *Microbacterium pumilum* 100%	** + **	** + **	**-**	** + + **
	B_IC3_V	Glossy white, irregularly shape	MN758847 *Rothia endophytica* 100%	**-**	** + **	**-**	** + **
	F_IC3_A3	Green–brown, hairy	KT832076 *Aspergillus medius* 97.56%	**-**	**-**	**-**	**-**
	F_IC3_A4	Green–brown, hairy	MT582752 *Aspergillus pseudoglaucus* 100%	**-**	**-**	**-**	**-**
IC7	B_IC7_A	Milky, circular	MH127765 *Pseudomonas vancouverensis* 99.84%	** + **	**-**	** + + **	** + + **
	B_IC7_A2	White-orange, flat	AY996839 *Nocardia* sp. 100%	** + **	**-**	** + **	**-**
	B_IC7_C	Yellow, circular, raised	MK670519 *Flavobacterium* sp. 99.17%	**-**	**-**	**-**	**-**
	B_IC7_F	Pink, slime	MT065733 *Ensifer adhaerens* 100%	**-**	**-**	** + + **	** + **
	B_IC7_H	Milky, opaque, circular	MK371085 *Pseudomonas* sp. 99.84%	**-**	**-**	** + + **	** + + **
	B_IC7_K	Orange, slime	MH698769 *Arthrobacter* sp. 100%	**-**	**-**	** + **	** + + **
	B_IC7_P	Milky, slime	MH337920 *Rhizobium* sp. 100%	**-**	**-**	** + + **	** + + **

-: no reaction displayed; + : positive reaction; +  + : extensive and/or rapid positive reaction

The denitrification capacity of the isolates and their ability to solubilize and precipitate CaCO_3_ and to oxidize ammonium are indicated in Table 3. Denitrification capacity was only observed in one fungal isolate (strain F_IC6_A1 *Purpureocillium* sp.). All activities tested, except sulfate oxidation activity, were demonstrated by the bacterial isolates although some differences were observed, i.e., the different bacteria displayed different activities/capacities. Several *Pseudomonas* and *Actinomycetes* (*Corynebacterium* sp., *Brevibacterium linens*, *Microbacterium pumilum*) isolates exhibited CaCO_3_ solubilization and precipitation, ammonium oxidation and denitrification capacities. Isolates belonging to the genus *Pseudomonas* and the classes *Actinomycetes*, *Bacilli*, and *Alphaproteobacteria* exhibited ammonium oxidation and denitrification capacities. Considering all bacterial isolates, 84% were capable of denitrification, 55% showed a positive reaction in the ammonia oxidation activity test, 26% solubilized CaCO_3_ and 18% formed CaCO_3_ crystals in B4 medium. Although several bacteria were isolated in *Thiobacillus* agar (medium specific for the isolation of sulfate-oxidizing bacteria), all of them displayed a negative reaction when bromocresol green was added as an indicator of sulfate-oxidizing activity.

### MinION sequencing analysis

All seven samples produced positive PCR results when the bacterial 16S rRNA and the fungal ITS fragments were targeted. The archaeal 16S rRNA was successfully amplified from samples IC9 and IC7. No archaeal DNA amplification or poor amplification was obtained in both samples of surrounding soil (ICS1 and ICS2). Amplification of the *nirK* gene (nitrite reductase/denitrifying bacteria) produced amplicons from samples IC6, IC7, and IC9. The *dsr* gene (dissimilatory sulfite reductase/sulfate-reducing bacteria) was detected in samples IC7 and IC9. The *soxB* gene (Sox enzyme system for sulfur oxidation/sulfur-oxidizing bacteria) was detected in samples IC6, IC7, and IC9 (Supplementary material Tables S1 and S2). The *amoA* gene (ammonia monooxygenase/ammonia-oxidizing bacteria) only produced results with the soil samples ICS1 and ICS2, and therefore these amplicons were not included in the MinION analysis. For characterization of the taxa, different minimum thresholds of relative abundance were established for amplicons for purposes of visualization. The reason for this was the different numbers of reads obtained for the different amplicon groups and the limited number of pre-defined reference sequences in the reference database utilized in EPI2ME Desktop Agent (ONT). After sequencing each run for 48 h, a total of 3,537,353 reads (first sequencing run, Supplementary Table S1) and 9,480,778 reads (second sequencing run, Supplementary Table S2) were obtained, with a total yield of 3.8 and 12.2 Gbases, respectively. The average quality scores were 10.15 and 8.78. The average sequence lengths were 984 and 1063 bases.

### Bacterial 16S rRNA sequencing evaluation

**Fig. 3:**
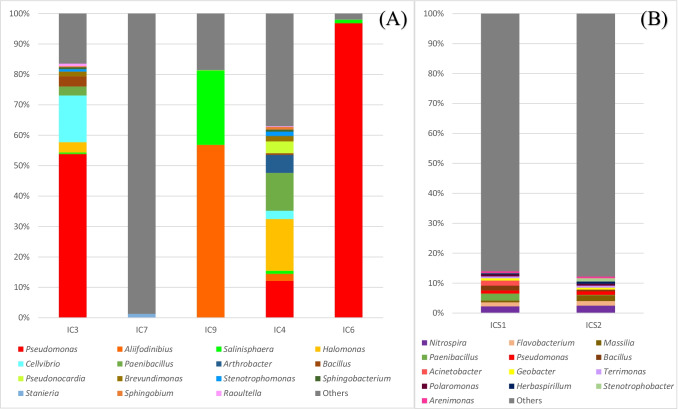
MinION sequencing of bacterial 16S rRNA gene of samples from the monastery (**A**) and from the surrounding soil (**B**). “Others” included all the taxa detected for which the sum of their percentages in all samples analyzed not reach higher than 1% (**A**) and 0.5% (**B**)

The results of the bacterial community analysis, performed by using long amplicons (about 1400 bp) that encoded the 16S rRNA gene, of the five samples from the monastery building (IC6, IC4, IC9, IC3, IC7) and the two samples from the surrounding soil (ICS1, ICS2) are summarized in Figs. 3A and B, respectively.

*Pseudomonas* spp. predominated in the bacterial communities from the interior environment especially in samples IC3, IC4, and IC6 (53.80%, 12.11%, and 96.83%, respectively, Fig. 3A). The second most abundant genus was *Aliifodinibius*, which dominated in sample IC9 (56.81%). This genus also occurred in sample IC4, but to a lesser extent (2.33%). The bacterial consortia in sample IC9 also included bacteria identified as *Salinisphaera* (24.42%). *Halomonas*, the 4th most abundant taxa detected, was mainly present in samples IC3 and IC4 (3.42% and 17.03%, respectively). The composition of the bacterial consortia in samples IC3 and IC4 were qualitatively similar. Other genera present in IC3 and IC4 included *Cellvibrio* (15.32% and 2.71%, respectively), *Paenibacillus* (2.96% and 12.43%), *Bacillus* (3.37% and 0.54%), *Brevundimonas* (1.55% and 1.79%), *Stenotrophomonas* (0.81% and 1.42%), *Sphingobacterium* (0.64% and 0.69%), and *Raoultella* (0.79% and 0.26%). The presence of these taxa was negligible in the other samples. *Arthrobacter* (5.92%) and *Pseudonocardia* (3.88%) were also detected in IC4.

The lowest bacterial diversity was observed in sample IC6, in which *Pseudomonas* predominated (96.83%)*.* The sample IC7 displayed the highest bacterial diversity; in fact, many different taxa were detected at a low abundance, e.g., the most abundant taxon in sample IC7 was the genus *Stanieria* (1.2%). “Others,” which includes all other taxa detected where the combined relative abundance in all samples did not reach higher than 1%, represented 16.48%, 98.73%, 18.44%, 37.01%, and 1.95% in samples IC3, IC7, IC9, IC4, and IC6, respectively.

Soil samples (ICS1 and ICS2) were characterized by a high diversity: many different taxa with low relative abundances (Fig. 3B). The five most abundant taxa of ICS1 and ICS2 were *Nitrospira* (2.25% and 2.51%, respectively), *Flavobacterium* (1.34% and 1.52%), *Massilia* (0.63% and 1.89%), *Paenibacillus* (2.30% and 0.17%), and *Pseudomonas* (1.02% and 1.18%). In the soil samples were not detected typical members of halophilic genera (*Aliifodinibius*, *Salinisphaera*, and *Halomonas*) which were abundant in several samples from the monastery. The high bacterial diversity caused an increase in the percentage of taxa included in the “Others” group. The reads assigned to given genera with relative abundance below 0.5% were included in this group. In samples ICS1 and ICS2, these taxa represented respectively 86.05% and 87.80% of all taxa present. Thus, the bacterial composition of both samples can be considered very similar.

### Fungal ITS sequencing

The sequencing of the fungal communities by ITS marker, performed with all 7 samples, generated a total of 505,096 reads. The results are graphically summarized in Supplemental Fig. S1.

The most abundant genera detected in samples IC3, IC7, IC9, IC4 and IC6 were as follows: *Laccaria* (3.29%, 16.23%, 6.38%, 26.97% and 4.39% in IC3, IC7, IC9, IC4 and IC6, respectively) followed by *Wickerhamomyces* (21.10%, 0.03%, 0.00%, 0.02%, 0.02%), *Batrachochytrium* (16.06%, 0.11%, 0.08%, 0.12%, 0.18%), *Botrytis* (1.99%, 2.62%, 5.30%, 0.04%, 6.27%), *Trichosporon* (4.56%, 0.46%, 1.24%, 3.29%, 2.02%), *Colletotrichum* (1.53%, 1.99%, 2.61%, 0.81%, 3.38%), *Dichomitus* (0.25%, 5.06%, 1.86%, 0.57%, 0.00%). *Phanerochaete* (2.90%, 0.74%, 1.35%, 2.38%, 0.00%), and *Penicillium* (1.79%, 1.87%, 0.59%, 0.36%, 0.55%). “Others” included the genera where the sum of their relative abundances in analyzed samples did not reach higher than 3%, with the following total abundances: 41.10%, 59.74%, 66.79%, 59.36%, and 74.81% in samples IC3, IC7, IC9, IC4, and IC6 respectively.

The fungal communities were similar in both soil samples (ICS1 and ICS2). The sequencing analysis detected members of the following genera, in both samples: *Linnemania*, *Podila*, *Pseudeurotium*, *Mortierella*, *Solicoccozyma*, *Mucor*, *Lipomyces*, *Cladosporium*, *Fibulochlamys*, *Exophiala*, *Saitozyma*, and *Juxtiphoma* (Supplemental Fig. S1B). “Others” (20.64% and 16.35% for ICS1 and ICS2, respectively) comprised those genera with a relative abundance below 2%.

### Archaeal 16S rRNA sequencing

**Fig. 4:**
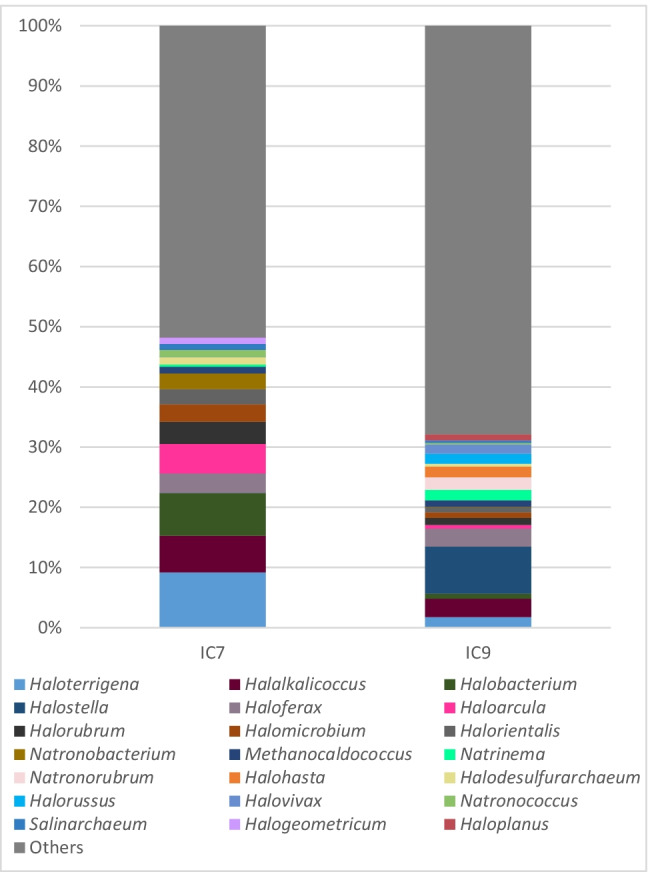
MinION sequencing of archaeal 16S rRNA. “Others” included all the taxa detected for which the sum of their percentages in all samples analyzed did not reach higher than 1%

The archaea detected in samples IC7 and IC9 mainly belonged to the class *Halobacteria*. The members of this class formed the majority of classified reads with relative abundances of 92.4% and 94.11% (for sample IC7 and IC9, respectively) and together with *Methanococci* (1.2% and 1.22% in IC7 and IC9, respectively), *Methanobacteria* (1.5% and 0.66%), and *Methanomicrobia* (0.5% and 041%) created almost all archaeal population identified by sequencing in these IC7 and IC9 samples. Three orders of the class *Halobacteria* were detected by sequencing analysis: *Halobacteriales* (29.27% and 34.57% in IC7 and IC9, respectively), *Natrialbales* (23.00% and 22.34%), and *Haloferacales* (8.39% and 9.39%). “Others” included the taxa where the combined relative abundance in the samples analyzed did not reach more than 1%, representing 51.76% and 67.8% in the samples IC7 and IC9, respectively. The genera of the archaea detected in this study and their relative abundance are graphically summarized in Fig. 4. The most commonly occurring genera identified in samples IC7 and IC9 were *Haloterrigena* (9.20% and 1.75%, respectively), *Halalkalicoccus* (6.08% and 3.05%), *Halobacterium* (7.10% and 0.88%), *Halostella* (0.00% and 7.81%), and *Haloferax* (3.26% and 2.95%).

### Denitrifying, sulfate-reducing and sulfur-oxidizing microbial communities

The sequencing analysis of *nirK* gene for denitrifying bacteria produced 15,936, 4581, and 3806 taxonomically classified reads for samples IC6, IC7, and IC9, respectively. The survey also revealed the presence of archaea and eukaryota. The reads from sample IC7 were composed by 76% bacteria, 13% archaea, 11% eukaryota, and less than 1% viruses. The microbial composition of sample IC9 was 63% archaea, 34% bacteria, and 3% eukaryota. Sample IC6 contained 99% bacteria and only 1% eukaryota.

**Fig. 5:**
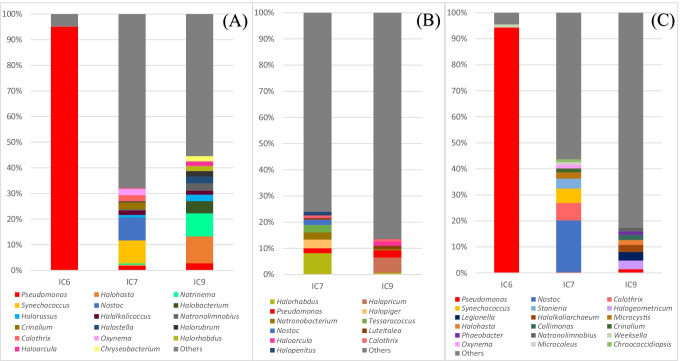
MinION sequencing of *nirK* gene, denitrifying bacteria and archaea (**A**); *dsr gene*, sulfate-reducing bacteria and archaea (**B**); and *soxB* gene, sulfur-oxidizing bacteria and archaea (**C**) of the monastery samples. “Others” included all the taxa detected for which the sum of their percentages in all samples analyzed did not reach higher than 2% (**A**), 1.5% (**B**), and 1% (**C**)

*Pseudomonas* (95.13%, 1.72%, and 2.79% in IC6, IC7, and IC9, respectively) was the most abundant genus detected in the analysis of denitrifying bacteria (Fig. 5A), especially in sample IC6, followed by *Natrinema* (archaea; 0.00%, 0.50%, and 8.99%), *Synechococcus* (0.03%, 8.95%, and 0.00%), *Nostoc* (0.00%, 8.80% and 0.00%), *Halobacterium* (archaea; 0.00%, 0.11%, and 4.78%), *Halorussus* (archaea; 0.00%, 1.03%, and 2.57%), *Halalkalicoccus* (archaea; 0.00%, 1.83%, and 1.45%), *Natronolimnobius* (archaea; 0.00%, 0.37%, and 2.89%), and *Crinalium* (0.00%, 2.75%, and 0.00%). The archaea *Haloarcula* was only detected in samples IC7 and IC9, with relative abundances of 0.28% and 1.81%, respectively. The study of denitrifying bacteria revealed the presence of *Cyanobacteria*, e.g., *Synechococcus* (8.95%), *Nostoc* (8.80%), and *Calothrix* (2.42%), mainly detected in sample IC7 (Fig. 5A). The relative abundances of the group “Others” was 4.82%, 67.85%, and 55.44% in the samples IC6, IC7, and IC9, respectively. The minimum cut-off for the “Others” was set at 2%.

Regarding the *dsr* gene used to study sulfate-reducing bacteria (SRB), 1,428,716 and 320,819 reads were obtained for samples IC7 and IC9, respectively. From those reads, only more than 1% (5414 and 1364, respectively) were classified. The most abundant taxa are summarized on the genus taxonomic level in Fig. 5B. The most abundant genera detected in the microbial analysis targeting SRB, with the exception of *Pseudomonas* (1.94% and 2.71% in IC7 and IC9, respectively), were archaea, e.g., *Halorhabdus* (8.13% and 0.66%), *Halapricum* (0.00% and 5.87%), *Halopiger* (3.29% and 0.00%), and *Natronobacterium* (2.73% and 0.51%). However, typical SRB was not detected by the sequence analysis. Other bacterial taxa detected in samples IC7 and IC9 include *Tessaracoccus* (2.81% and 0.00%) and *Nostoc* (2.03% and 0.00%). “Others” reached abundances of 76.01% and 86.29% in IC7 and IC9, respectively, with the threshold of relative abundance established at 1.5%.

The *soxB* gene of sulfur-oxidizing bacteria (SOB) was only amplified from samples IC6, IC7, and IC9. The results of the sequencing analysis are summarized in Fig. 5C. Almost all of the reads from sample IC6 belonged to the genus *Pseudomonas* (94.29%). The most abundant taxa in sample IC7 were *Nostoc* (19.87%), followed by *Calothrix* (6.73%), *Synechococcus* (5.56%), *Stanieria* (3.80%), and *Microcystis* (2.39%). These genera were detected in the other samples (IC6 and IC9) with negligible relative abundance or not detected at all. The genera *Halogeometricum*, *Legionella*, *Halalkaliarchaeum*, and *Halohasta* occurred only in sample IC9 with relative abundances of 3.32%, 3.29%, 2.73%, and 1.92%, respectively. “Others” included the taxa for which the relative abundance in given samples did not reach higher than 1%. The abundances of these taxa were 4.47%, 56.29%, and 82.61% in sample sIC6, IC7, and IC9, respectively.

## Discussion

The analysis of water-soluble ions (anions and cations) indicated the potential presence of highly soluble salts, such as calcium nitrate and magnesium nitrate, together with the most insoluble salts, such as calcium sulfate—gypsum (Freedland 1999; Steiger 2016). Based on the presence of this combination of salts, it is possible that the damp to touch dark staining is caused by the gypsum (poorly soluble) changing the size or filling the pores of granite and leading to an increased susceptibility to water condensing on the stone, forming a thin film of water in which highly soluble salts could dissolve. This would also explain why the presence of sulfates is triggered when nitrates are eliminated by bio-desalination, as in sample IC4 (Table 2). Nonetheless, the salt contents in the areas analyzed inside the building cannot fully explain the presence of the damp dark staining (Table 1). Indeed, in sample IC7, with no staining, the total ion content (57.958 mg/g rock) was higher than in samples IC9 (13.972 mg/g rock) and IC3 (16.820 mg/g rock) on which intense dark staining was observed (Table 1). Likewise, damp dark patches were much more visible in sample IC3, which had a lower content of salt ions (Table 1) than IC4, which had about ten times as much total ion content (107.248 mg/g rock). There is, therefore, no conclusive explanation of what exactly causes the damp dark staining, and further analysis in relation to organic matter and recalcitrant elements present in the stonework is required.

This research has shown, for the first time, the usefulness of third-generation sequencing (MinION) for identifying the microbiota in salt-contaminated granite stone. It is also the first time that the complete microbiota on stonework treated by bio-desalination has been identified by MinION sequencing. Furthermore, to our knowledge, the combination of MinION sequencing and some PCR assays (using the primers Arc344f-mod/Arch958r-mod, nirKC1F/nirKC1R, DSR1deg/DSR4Rdeg, and soxB432F/soxB1446B) was also applied in this study for the first time. The MinION-based approach allowed sequencing of different sizes of amplicons from around 500–600 bp for the gene *nirK* and archaeal 16S rRNA; the long fragments of about 1000, 1400, and 1900 bp of the *soxB*, bacterial 16S rRNA, and *dsr* gene, respectively.

The study findings revealed that bacterial and fungal communities, as well as the type and content of salt present in the monastery building, are very diverse and different from those in the surrounding soil (Fig. 2, Fig. 3, Supplemental Fig. S1 and Table 2). A common characteristic of almost all samples from inside the building (and absent outside) was the large amounts of soluble salts detected and the occurrence of halophilic bacteria such as *Aliifodinibius* and *Halomonas* (Table 2 and Fig. 3). The genus *Aliifodinibius* comprises species that are mainly isolated from salty environments: *A. roseus* and *A. sediminis* were isolated from salt mine samples (Wang et al. 2013), *A. halophilus* (Xia et al. 2016) from marine solar salterns and *A. salicampi* from gray salterns (Cho et al. 2017). In previous studies, members of the genus *Salinisphaera* were isolated directly from seawater (*S. aquimarina* and *S. dokdonensis*; Tang et al. 2018; Bae et al. 2010) or seawater environments (*S. orenii* and *S. japonica*; Park et al. 2012; Shimane et al. 2013). *Halomona*s spp. also belong to halophilic bacteria and *H. muralis* was isolated, for the first time, from a biofilm colonizing the walls of the Saint Catherine Chapel in Austria (Heyrman et al. 2002). Furthermore, microorganisms involved in nitrate and sulfate cycles were detected in almost all samples taken from the building, which is consistent with the large amounts of nitrates and sulfates detected in the samples (Table 2, Table 3, and Fig. 5). The denitrifying bacteria were mainly represented by *Pseudomonas*, whose presence was confirmed in all PCR positive samples, especially in the partially bio-desalinated sample IC6, but also in the treated by bio-desalination samples IC3 and IC4 (Fig. 5A). It is clear, using the culture-independent MinION sequencing, the dominance of the artificially introduced bacterium *P. stutzeri* in those sampling places (mainly in IC6), which, however, is not detected at that level with the culture-dependent analysis, in agreement with the results of Laiz et al. (2003). In this regard, although in most biocleaning treatments the strain used is completely removed after treatment (see, for example, Ranalli et al. 2005 and documents citing this publication), a few cases have been reported where the bacterial cells are left on the cleaned surface to continue to function (Cappitelli, pers. comm.). Here, the bacterial cells of *P. stutzeri* have been removed with water and sponge and their non-presence has been checked by CFU counting (see the “Sampling” section), in spite of which they have remained in the treated areas, continuing their nitrate biocleaning function. This is indicated by the appearance of struvite in the sample IC6 (see the “Salt content” section) and the significant reduction in nitrate levels one year after biocleaning (see Bosch-Roig et al. 2021). The reasons that have probably favored this are the high humidity of the chapel paving slabs (damp to touch), because the maintenance of the cells for biocleaning is achieved by keeping the humidity in the treatment area (see, for example, Bosch-Roig and Sanmartín 2021), as well as the movement that the *P. stutzeri* cells may have towards the inside of the granite stonework, looking for nitrates and other possible organic materials (Lalucat et al. 2006; Bosch-Roig et al. 2016). The latter would prevent *P. stutzeri* cells from being eliminated in the final step with water and sponge of the biocleaning process described above, which only affects the surface. Therefore, the positive consequences of the introduction of this bacterium in an autochthonous community from a salt-contaminated substrate (as is the case here) are continuing to perform biocleaning of nitrates and possible organic substances that contaminate the paving. Although it is obligatory to monitor, measure and document its presence in the coming months, since (and these are the negative aspects) these foreign cells may interact with the substrate and also increase fungal presence. In this sense also indicate that the microbiota of the paving altered by *P. stutzeri* can be restored naturally (without having to remove the *Pseudomonas* cells) after some time, as has been reported in other works such as Ettenauer et al. (2011), where an exogenous species (*Mixococcus xanthus*) was introduced for biotrearment dominating the microbiota but one year after the microbiota was partly re-established.

Some of the isolated strains also showed the ability to precipitate and/or solubilize CaCO_3_ (see Table 3). This concurs with the findings of other studies that have linked the nitrate reduction activity of different denitrifying bacteria to their ability to precipitate CaCO_3_ (Lin et al. 2021). Furthermore, the presence of denitrifying, sulfate-reducing archaea and ammonification microorganisms could potentially degrade the other organic materials present on the granite stonework, leading to stone corrosion as described by other authors (Zhang and Zhang 2006; Li et al. 2012).

The strong presence of *Actinobacteria* (Fig. 2), detected in and isolated from sample IC4, belonging to genera such as *Arthrobacter*, *Pseudonocardia* and *Corynebacterium* is also interesting (Fig. 3 and B_IC4_N in Table 3). This is consistent with reports by other authors who found actinobacteria on salt-contaminated stone monuments (Laiz et al. 2009). In the present case, actinobacteria may be related to the presence of gypsum (Table 2) because they are able to precipitate this mineral (Cirigliano et al. 2018; Trifi et al. 2020). Moreover, the greater occurrence of some species of *Firmicutes* (*Paenibacillus* and *Bacillus*, e.g., in isolate B_IC4_E in Table 3) may also contribute to the precipitation of gypsum (Kinnunen et al. 2020). In this sense, in addition to the bacteriogenic origin, an anthropogenic origin cannot be ruled out since gypsum as coating has been historically used in many stone buildings.

Several members of the archaea, detected in the samples IC7 and IC9, seemed to be indigenous microbiota. They belong to the class *Halobacteria* and members of the genera *Haloterrigena*, *Halalkalicoccus*, *Halobacterium*, *Halostella*, and *Haloferax*. It seems that only the genera *Halalkalicoccus* and *Halobacterium* were already detected in salt efflorescences present on the stone surfaces of several churches in Austria (Ettenauer et al. 2010; 2014). Nonetheless, the genera *Haloterrigena*, *Halostella*, and *Haloferax* are considered extremely halophilic (Gutiérrez et al. 2008; Song et al. 2016) and also members of obligate halophilic archaea (Haque et al. 2020).

Several archaea were detected by sequencing using PCR assays oriented to the denitrifying, sulfate-reducing, and sulfite-oxidizing bacteria (mainly in pavement sample IC9, but also in wall sample IC7). This could be due to the similarity of the genes *nirK* (Bartossek et al. 2010) and *dsr* (Wagner et al. 1998) in bacteria and archaea. Together with the *Halobacteria* described above, other denitrifying archaea were detected (*nirk* gene sequencing): *Halohasta*, *Natrinema*, *Halorussus*, *Natronolimnobius*, *Halorubrum*, *Halorhabdus*, and *Haloarcula*. The denitrifying capacities of these haloarchaea were recently reviewed in relation to removal of pollutants from wastewater (Li et al., 2022). The denitrifying *Chryseobacterium* was only found in sample IC9, and nitrogen-fixing *Cyanobacteria* were very abundant in the wall sample IC7, in which the concentration of salts was highest. The greater presence of cyanobacteria on the stone wall than on the paving slab may be due to diverse availability of water, light, and pH, as other authors have demonstrated (Rego et al. 2019). These phototrophic salt-tolerant microorganisms are common colonizers of historic stone surfaces (Miller and Macedo 2006).

The *dsr* and *soxB* studies revealed different trends regarding microbes involved in the sulfate cycle (Fig. 5). Differences in the presence of *Pseudomonas* (mainly in IC6), cyanobacteria (almost exclusive in IC7), and archaea (in IC7 and IC9) were observed. The detection of sulfate reducers (by the *dsr* gene, Fig. 5B) revealed that high percentages of the other genera of archaea (*Halapricum*, *Halopiger*, and *Halopenitus*) were not detected by the other gene markers. These results are consistent with recent findings involving sulfur respiration in halophilic archaea (Sorokin et al. 2018; 2021). The *dsr* approach specific for sulfate-reducing bacteria failed to detect these bacteria. It seems that the high concentration of salts of the samples provided suitable conditions for the growth of the halophilic archaeal community rather than other groups of bacteria.

In sample IC9, among the sulfur oxidizers (with high percentages of reads), the bacterial genera *Legionella*, *Collimonas*, and *Phaeobacter* appeared together with archaea. Sulfur-oxidizing microorganisms on stone can reduce CO_2_ as a carbon source by oxidizing H_2_S or elemental sulfur to sulfurous or sulfuric acid that can dissolve the minerals in stone (Wang and Liu 2021). In this case, the salt conditions of the studied environment permitted the proliferation of haloarchaea, which substituted the canonical sulfur-oxidizing bacteria in the sulfur oxidation cycle. Moreover, the occurrence of several *Legionella* species in an environment containing high levels of sulfur has previously been reported (Sheehan et al. 2005). The presence of the genes for the sulfur-oxidizing enzyme subunits SoxCD in *Phaeobacter* members suggests their potential ability to oxidize sulfur compounds (Vietti 2014). *Collimonas* spp. are common in oligotrophic habitats and are responsible for mobilizing iron, and the mineral weathering potential has therefore been suggested as a functional characteristic of this genus (Uroz et al. 2009). No link between *Weeksella* species, detected in sample IC6, and stone deterioration or oxidation of sulfur compounds has been found.

Unfortunately, PCR amplification of the bacterial *amoA* gene did not produce amplicons from the building samples. One possible reason why ammonium-oxidizing bacteria could not be isolated and amplified from granite surfaces is that the archaea are the main ammonia oxidizers on stone surfaces affected by high salinity (Meng et al. 2017). Thus, only the culture-dependent approach was able to show the presence of ammonium-oxidizing bacteria in the samples analyzed. The ammonium oxidizers mainly belonged to the genera *Pseudomonas*, *Rhizobium*, *Streptomyces, Paenarthrobacter*, and *Ensifer*. Nitric and nitrous acids are produced on stone surfaces by ammonia- and nitrite-oxidizing microorganisms, including heterotrophic bacteria. Such microbial communities lead to the weakening of the stone matrix and consequently are the cause of stone decay (Scheerer et al. 2009).

The fungal communities of the indoor samples, considering the most commonly detected fungi, were composed of the same genera (Supplemental Fig. S1). Members of the genus *Laccaria* may be involved in weathering minerals and metals (Fomina et al. 2006; Uroz et al. 2009). In addition, the extraction of various minerals is more effective when *Laccaria* species are associated with *Pseudomonas* species (Uroz et al. 2009). According to the strong presence of the genera *Laccaria* and *Pseudomonas* in all samples examined in the present study, this type of symbiosis will probably occur in the study environment.

Sample IC3 was also characterized by the presence of the genera *Wickerhamomyces* and *Batrachochytrium*. It is difficult to establish a connection between these two genera and the given environment as the first genus is a food-associated fungus (Tofalo et al. 2020), while the second is a known pathogen of amphibians (Rollins-Smith 2020). The *Batrachochytrium* presence in the chapel paving attracts attention because *Batrachochytrium dendrobatidis* is causing a massive decline in global amphibian populations (Daszak et al. 2003) and amphibian chytridiomycosis caused by this fungus and other species of *Batrachochytrium* has been considered the most devastating disease of all vertebrate groups (Berger et al. 1998). Despite species of *Batrachochytrium* being described as a saprophyte, to the best of our knowledge, this is the first time it has been found in a building. Rural buildings are sometimes visited by amphibians and humans could be dispersing the dangerous pathogen too just by step on it and walk. A further search of the building and also monitoring will be carried out in this regard.

Fungi belonging to the genera *Botrytis*, *Trichosporon*, and *Colletotrichum* were detected in all samples and their involvement in stone and stonework deterioration has also been shown in previous studies (Urzì et al. 2010; Gaylarde et al. 2017; Trovão et al. 2019). Some fungi such as *Dichomitus* and *Phanerochaete* have displayed the ability to degrade different natural polymers (e.g., lignin; Conesa et al. 2002) by the production of extracellular enzymes that attack polycyclic aromatic hydrocarbons (PAHs; Wang et al. 2009; Covino et al. 2010).

In conclusion, the study findings showed that the most abundant anions present in the monastery building, i.e., nitrate (NO_3_^−^) and sulfate (SO_4_^2−^), are associated with the microbial communities detected, i.e., denitrifying, ammonia-oxidizing, sulfate-reducing, and sulfate-oxidizing bacteria. Although no direct relationship was found between the damp-to-touch dark staining and the salt content and the related microbiota (necessitating further analysis in relation to organic matter and recalcitrant elements in the stonework), a well-defined relationship was found between the microbes and the soluble salts present. The state-of-the-art technology used for microbial characterization yielded findings regarding the microbial diversity and phylogenetic groups present and also helped us to gain some insight into the biological cycles occurring in the community and the organisms and key genes involved in the different geomicrobiological cycles.

## Supplementary Information

Below is the link to the electronic supplementary material.

Supplementary file1 (PDF 353 KB)

## Data Availability

All data generated or analyzed during this study are included in this publication (and the supplementary material).
