# Supplementary material for: Long-amplicon MinION-based sequencing study in a salt-contaminated twelfth century granite-built chapel
Source: Appl Microbiol Biotechnol. 2022 May 21;106(11):4297–314. doi: 10.1007/s00253-022-11961-8 (PMC9200699; doi:10.1007/s00253-022-11961-8)
Supplement: Supplementary file 1 — Supplementary file1 (PDF 353 KB) [file 253_2022_11961_MOESM1_ESM.pdf]

Supplementary Material

Long-amplicon MinION-based sequencing study in a salt-contaminated 12th century granite-built chapel

Jelena Pavlović[a,1], Pilar Bosch-Roig[b,1], Magdalena Rusková[a], Matej Planý[a], Domenico Pangallo[a,c], Patricia Sanmartín[d,e,*]

[a] Institute of Molecular Biology, Slovak Academy of Sciences, Dúbravská cesta 21, 845 51 Bratislava, Slovakia. jelena.pavlovic@savba.sk; magdalena.kapustova@savba.sk; matej.plany@savba.sk;

[b] Instituto Universitario de Restauración del Patrimonio. Universitat Politècnica de València, 46022, Valencia, Spain. mabosroi@upvnet.upv.es ORCID: 0000-0003-2019-399X

[c] Caravella, s.r.o., Tupolevova 2, 851 01 Bratislava, Slovakia. domenico.pangallo@savba.sk ORCID: 0000-0002-9115-9409

[d] Departamento de Edafoloxía e Química Agrícola, Facultade de Farmacia. Universidade de Santiago de Compostela, 15782, Santiago de Compostela, Spain. patricia.sanmartin@usc.es ORCID: 0000-0002-5733-8833

[e] CRETUS, Universidade de Santiago de Compostela, Santiago de Compostela, Spain.

* Corresponding author: patricia.sanmartin@usc.es (Patricia Sanmartín)

[1] Both authors contributed equally to this work

Fig. S1 MinION sequencing of fungal ITS fragment of samples from church (A) and from outdoor soils (B). “Others” included all the taxa detected for which the sum of their percentages in all analyzed samples did not reach a relative abundance higher than 3% (A) and 2% (B).

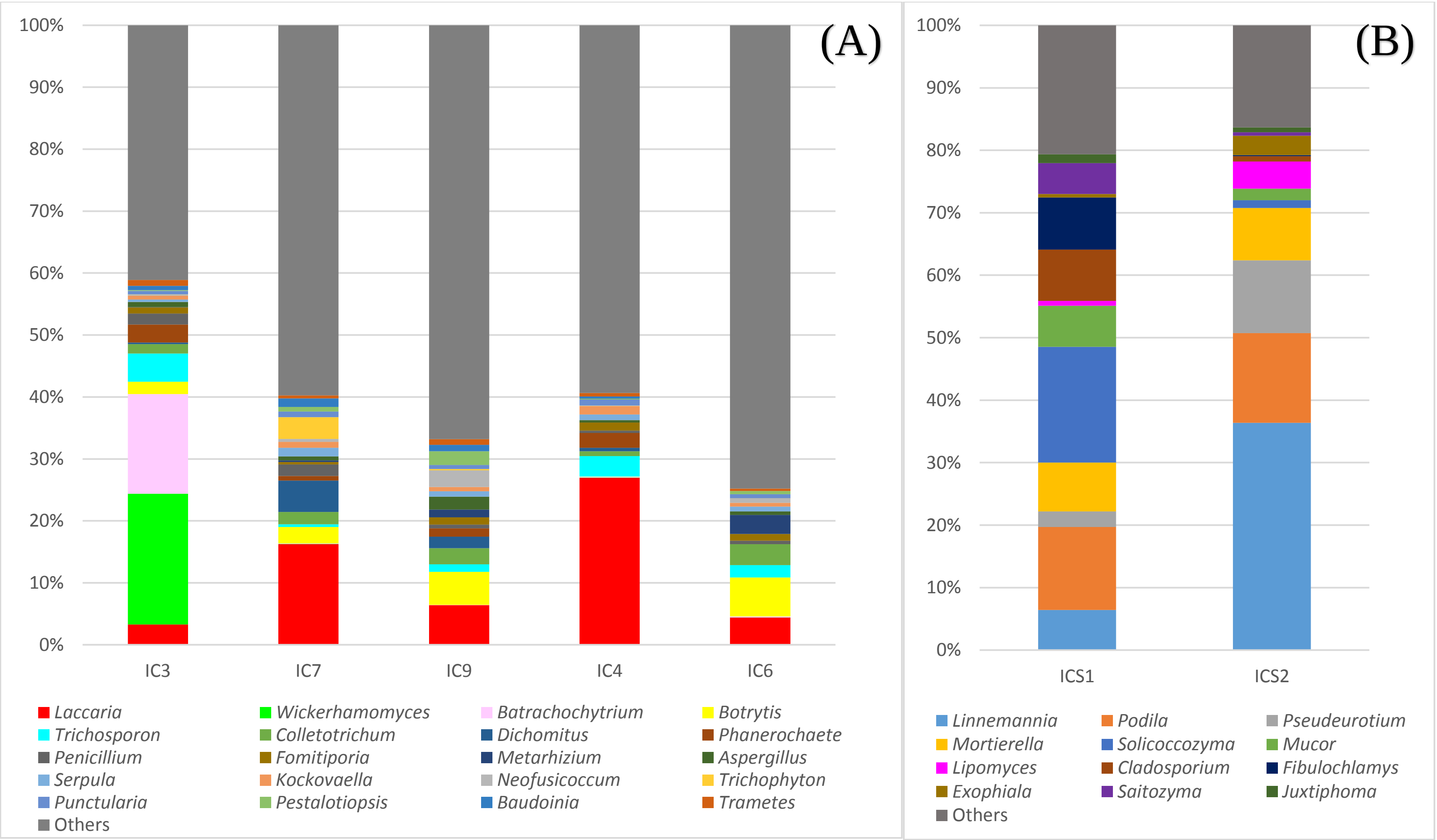

Table S1 First sequencing run with 3537353 reads analysed, with the total yield of 3.8 Gbases, 10.15 average quality score, and 984 average sequence length.

Sequencing library	Sample	Amplicon	Number of reads	Classified reads	Unclassified reads
1	IC3	ITS	79270	44831	32367
2	IC7	ITS	52805	12486	38778
3	IC9	ITS	5080	3716	1209
4	IC4	ITS	27997	19369	7739
5	IC6	ITS	6967	4561	1984
6	IC7	Archaeal 16S rRNA	145535	113137	28267
7	IC9	Archaeal 16S rRNA	274414	216536	51118
8	IC3	Bacteria 16S rRNA	153262	148756	1104
9	IC7	Bacteria 16S rRNA	903031	860152	25644
10	IC9	Bacteria 16S rRNA	572667	533032	15611
11	IC4	Bacteria 16S rRNA	424773	404614	6068
12	IC6	Bacteria 16S rRNA	891552	861780	5982

Table S2 Second sequencing run with 9480778 reads analysed with the total yield of 12.2 Gbases, 8.78 average quality score, and 1063 average sequence length.

Sequencing library	Sample	Amplicon	Number of reads	Classified reads	Unclassified reads
1	ICS1	Bacterial 16S rRNA	3256417	968896	150124
2	ICS2	Bacterial 16S rRNA	2645776	842842	108268
3	ICS1	ITS	219457	28896	65423
4	ICS2	ITS	113520	19402	32175
5	IC6	*nirK* gene	124963	15936	43182
6	IC7	*nirK* gene	509665	4581	218401
7	IC9	*nirK* gene	193151	3806	79573
8	IC6	*soxB* gene	359845	99044	39406
9	IC7	*soxB* gene	198123	29623	159667
10	IC9	*soxB* gene	110326	3438	41872
11	IC7	*dsr* gene	1428716	5414	515829
12	IC9	*dsr* gene	320819	1364	127996

Table S3 Particle size distribution of the soil samples.

	Clay (%)	Fine silt (%)	Coarse silt (%)	Fine sand (%)	Coarse sand (%)
**ICS1**	6.9	13.0	7.7	25.8	46.6
**ICS2**	7.5	14.8	8.2	24.4	45.0
